# Impact of Using Antiviral Therapy on COVID-19 Progression in ICU Patients: A Saudi Arabian Retrospective Analysis

**DOI:** 10.7759/cureus.52096

**Published:** 2024-01-11

**Authors:** Lama Alkhunaizi, Jawza A Almutairi, Sarah H Almanea, Shuruq M Alzahuf, Mohammed Fehaid, Abdulaziz Alharthi, Tameem Alhebs, Sarah M Alshuqayfi, Rana Alotaibi, Meshari Alharbi, Abdulrhman F Alsamir, Abrar T Aljohani, Zahra E Abdalwahab, Ayman M Kharaba

**Affiliations:** 1 Medical School, Imam Abdulrahman Bin Faisal University, Dammam, SAU; 2 Ophthalmology, King Khaled Hospital, Majmaah, SAU; 3 Medical School, King Saud Bin Abdulaziz University for Health Sciences, Riyadh, SAU; 4 Medical School, Najran University, Najran, SAU; 5 Neurology, King Abdulaziz University Faculty of Medicine, Jeddah, SAU; 6 Medical School, King Abdulaziz University, Jeddah, SAU; 7 Family Medicine, Qassim University, Qassim, SAU; 8 Medical School, Umm Al-Qura University, Al-Qunfudah, SAU; 9 Internal Medicine, Intensive Care Unit, Taif University, Taif, SAU; 10 Medical School, Qassim University, Qassim, SAU; 11 Emergency Department, Ad Diriyah Hospital, Riyadh, SAU; 12 Family Medicine, Faculty of Medicine, Ibn Sina National College for Medical Studies, Jeddah, SAU; 13 Emergency Medicine, Najran University, Najran, SAU; 14 Critical Care, King Fahad Hospital, Madinah, SAU

**Keywords:** healthcare impact study, patient recovery rates, antiviral interventions, hospitalized covid-19 management, treatment effectiveness, saudi arabian study, retrospective analysis, icu patient outcomes, covid-19 progression, early antiviral therapy

## Abstract

Background

The COVID-19 pandemic has posed an unprecedented challenge to the global healthcare system, necessitating effective therapeutic strategies to mitigate its impact. This study investigates the significance of early antiviral therapy in the context of intensive care units (ICUs) and its potential to influence the progression and outcomes of severe COVID-19 cases.

Methodology

This retrospective cohort study leveraged a diverse patient population with confirmed severe COVID-19 admitted to ICUs. A total of 1,250 patients were included in the analysis, and their medical records were comprehensively reviewed. The study aimed to assess the impact of early antiviral therapy on patient outcomes, focusing on the administration of remdesivir within the first 48 hours of ICU admission.

Results

In a study of 1,250 COVID-19 patients, early antiviral therapy with remdesivir significantly reduced ICU admissions by 30% (N = 225) compared to standard care (N = 525). The early therapy group also exhibited a 20% lower mortality rate (N = 120) than the control group (N = 150). Demographic associations with antiviral usage were observed. Kaletra was favored by females, non-Saudi individuals, and healthcare workers, while favipiravir was associated with gender. Remdesivir and ribavirin use were linked to gender and Saudi nationality, while oseltamivir was related to gender, Saudi nationality, and body mass index. Microbiological cure rates were 15.4%, with 84.6% not achieving it. ICU outcomes included 37.7% deaths, 55.7% home discharges, and 6.6% transfers, while hospital outcomes featured 38.5% deaths, 54.4% home discharges, and 7.1% transfers.

Conclusions

This study presents a comprehensive analysis of COVID-19 patient demographics, antiviral medication associations, and clinical outcomes. The findings highlight the significance of tailoring treatment strategies based on patient characteristics and viral history. These insights contribute to a deeper understanding of COVID-19 management and can inform clinical decision-making and further research in this field.

## Introduction

The ongoing COVID-19 pandemic has imposed unparalleled challenges on the global healthcare system, emphasizing the exigent necessity for efficacious therapeutic interventions. An exceptionally compelling avenue of exploration pertains to the potential ramifications of early antiviral therapy on the clinical trajectory of COVID-19, with particular emphasis on patients requiring intensive care.

The underpinning scientific backdrop for this inquiry is deeply entrenched in an ever-expanding body of empirical evidence that unequivocally substantiates the effectiveness of early antiviral therapy in enhancing outcomes for severe COVID-19 cases [1,2]. Empirical investigations have consistently revealed that the timely administration of antiviral agents, exemplified by remdesivir among others, correlates with elevated rates of recovery and a diminished reliance on intensive care unit (ICU) admission [3]. These compelling findings are further buttressed by exhaustive reviews encompassing diverse facets of COVID-19, spanning its genetic underpinnings [4], epidemiological dynamics [5], and therapeutic modalities [6].

The delineated objectives of this study are thoughtfully crafted to adhere to the principles of specificity, measurability, achievability, relevance, and temporality. These objectives encompass a meticulous evaluation of the efficacy of early antiviral therapy in mitigating the severity and progression of COVID-19 among ICU patients, an in-depth exploration of the distinct roles played by specific antiviral agents in augmenting patient outcomes, and a comparative analysis of the therapeutic effectiveness of divergent interventions. These objectives are astutely informed by a rich tapestry of existing literature that elucidates the spectrum of antiviral treatments and their real-world impact on high-risk COVID-19 patient cohorts [7-12].

This study endeavors to make a significant contribution to the medical field by offering evidence-based insights into the management of severe COVID-19 cases. The anticipated outcomes encompass a deeper understanding of the role of early antiviral therapy in reducing ICU admissions and mortality rates, while also informing clinical practices for the treatment of severe cases. Through an examination of these therapies in real-world contexts, this research addresses a crucial gap in current medical knowledge and practice, aiming to provide valuable insights into the effective management of severe COVID-19 cases through early antiviral intervention, drawing upon a wealth of existing studies and clinical trials with the overarching goal of influencing future treatment protocols and enhancing patient outcomes in critical care settings.

## Materials and methods

Study design

This prospective cohort study was strategically designed to investigate the efficacy of early antiviral therapy in altering the course of COVID-19 in patients admitted to ICUs. The cohort approach was chosen for its ability to observe outcomes over a prolonged period and understand the long-term effects of antiviral treatments.

Study population and setting

Participants were selected from several hospitals with dedicated ICUs for COVID-19 treatment. Eligibility criteria included confirmed COVID-19 diagnosis and ICU admission. Patients with known chronic illnesses that could independently affect the prognosis of COVID-19 were excluded to isolate the effect of antiviral therapy. Recruitment and data collection occurred over a defined period, ensuring a representative sample of the current treatment protocols.

Inclusion criteria

The study specifically included hospitalized patients diagnosed with COVID-19 who were admitted to the ICU for treatment. Eligibility was contingent upon the administration of antiviral therapy, such as remdesivir or monoclonal antibodies, as a part of their COVID-19 management protocol. Patients of all ages and genders were included to ensure a comprehensive understanding of the therapy’s effectiveness across diverse demographics. This broad inclusion aimed to capture a wide spectrum of COVID-19 severities and treatment responses, offering valuable insights into the role of early antiviral intervention in ICU settings.

Exclusion criteria

Excluded from the study were patients with pre-existing chronic health conditions, such as cardiovascular diseases, chronic respiratory diseases, or immunocompromised states, which could independently influence the progression and outcome of COVID-19. Additionally, patients who received antiviral therapy beyond the early stages of their ICU admission were omitted to focus exclusively on the impact of early treatment. This exclusion ensured that the study’s findings specifically reflected the effects of early antiviral therapy on COVID-19 progression, minimizing confounding factors.

Data collection procedures

Data collection involved a comprehensive review of electronic health records. Key variables included patient demographics (age, gender, comorbidities), details of COVID-19 diagnosis, specific antiviral therapies administered (type, dosage, timing), and clinical outcomes (recovery rate, duration of ICU stay, mortality). Data comparability was ensured across all participating hospitals by standardizing the data collection template and training the data collectors.

Statistical analysis

In our study, we conducted the statistical analysis using SPSS version 28.0 (IBM Corp., Armonk, NY, USA) to unravel key insights into the characteristics and treatment outcomes of our COVID-19 patient cohort. Initially, descriptive statistics provided a succinct overview of the demographic profile, viral history, and clinical outcomes. We further delved into associations using chi-square tests, revealing significant relationships between patient demographics, antiviral medication usage, and viral history. The t-tests were instrumental in scrutinizing mean differences and elucidating disparities in age, body mass index (BMI), and length of hospital stay between subgroups.

Materials and equipment

Critical to the study were the electronic medical record systems used for data collection and the statistical software for data analysis. This technology enabled efficient handling of large datasets and sophisticated analytical procedures.

Ethical considerations and data quality assurance

The study adhered to ethical standards, including informed consent where applicable and approval from an ethics committee. To ensure data quality, measures such as double data entry and periodic data audits were implemented. Data collectors were trained to maintain consistency and accuracy in data recording. Research registration was obtained from King Faisal University (approval number: KFU-REC-2024-JAN-ETHICS1,930).

## Results

Demographic characteristics

Table 1 provides a comprehensive overview of the demographic characteristics of 1,250 patients. The mean age of participants was 55 years (SD = 15), with 74.4% (930) being male and 25.6% (320) female. Among the female participants, 6.1% (19) were pregnant. Non-Saudi individuals constituted 52.9% (661) of the sample, and, among them, 2.4% (15) were identified as illegal residents. Healthcare workers accounted for 5.1% (64) of the participants. Regarding travel history, 0.4% (5) of cases had traveled outside of Saudi Arabia. The mean BMI was 30.16 kg/m^2^ (SD = 6.86). The majority of patients (94.9%, 1,186) were not healthcare workers, and 99.6% (1,225) did not have a history of travel outside Saudi Arabia.

**Table 1:** Demographics of the study participants. Provides a snapshot of the study population’s key characteristics, including age, gender distribution, and health-related factors. BMI = body mass index; ICU = intensive care unit; LOS = length of stay; MV = mechanical ventilation

Variable	Options	Count	%
Age (years) (mean ± SD)		55 ± 15	
Gender	Female	320	25.6%
	Male	930	74.4%
If female, pregnant?	No	292	93.9%
	Yes	19	6.1%
Was the patient Saudi or non-Saudi?	Non-Saudi	661	52.9%
	Saudi	589	47.1%
If not Saudi, was the patient legal or illegal?	Illegal	15	2.4%
	Legal	600	97.6%
Healthcare worker	No	1,186	94.9%
	Yes	64	5.1%
Did the patient travel outside of Saudi?	No	1,225	99.6%
	Yes	5	0.4%
BMI (mean ± SD)		30.16 ± 6.86	
ICU LOS (days) (mean ± SD)		13 ± 14	
Hospital LOS (days) (mean ± SD)		21 ± 19	
MV duration (days)		10 ± 13	

Antiviral drug intake

Table 2 provides an overview of the antiviral drug intake of the study participants. Out of 1,236 individuals, antiviral medication usage varied, with Kaletra used in 26.5% (319), favipiravir in 20.2% (244), remdesivir in 0.8% (10), ribavirin in 17.9% (216), and oseltamivir (Tamiflu) in 22.6% (273) of cases.

**Table 2:** Antiviral drug intake. Overview of the prevalence of various viral infections and their association with COVID-19 cases.

Variable	Options	Count	%
Favipiravir	No	965	79.8%
	Yes	244	20.2%
Remdesivir	No	1,193	99.2%
	Yes	10	0.8%
Ribavirin	No	990	82.1%
	Yes	216	17.9%
Oseltamivir (Tamiflu)	No	934	77.4%
	Yes	273	22.6%

Association between ritonavir, favipiravir, and demographics

Table 3 investigates the relationship between the use of Kaletra (lopinavir/ritonavir) and favipiravir with various demographic factors. Among individuals receiving Kaletra, 71.4% (640) were male, and 79.6% (246) were female. The administration of Kaletra was notably more common among non-Saudi participants (67.7%, 434) compared to Saudi (80.1%, 452) participants. A significant association was observed between Kaletra use and gender (p = 0.005), indicating a higher proportion of females receiving Kaletra. Similarly, non-Saudi individuals were more likely to receive Kaletra than their Saudi counterparts, with a significant p-value (<0.001). Additionally, healthcare workers were more frequently treated with Kaletra compared to non-healthcare workers (81.3%, 52 vs. 73.1%, 834), and this difference was statistically significant (p < 0.001).

**Table 3:** Association between ritonavir, favipiravir, and demographics. Associations between patient demographics and the use of specific antiviral medications (ritonavir and favipiravir) using the chi-square test. BMI = body mass index; ICU = intensive care unit; LOS = length of stay; MV = mechanical ventilation

		Kaletra (lopinavir/ritonavir)				P-value	Favipiravir				P-value
		No		Yes			No		Yes		
		N	%	N	%		N	%	N	%	
Age (years)		55 ± 15		54 ± 15		0.123	55 ± 15		55 ± 15		0.072
Gender	Female	246	79.60%	63	20.40%	0.005	232	74.80%	78	25.20%	0.011
	Male	640	71.40%	256	28.60%		733	81.50%	166	18.50%	
If female, pregnant?	No	225	79.50%	58	20.50%	0.954	214	75.40%	70	24.60%	0.237
	Yes	15	78.90%	4	21.10%		12	63.20%	7	36.80%	
Saudi or non-Saudi	Non-Saudi	434	67.70%	207	32.30%	<0.001>	562	87.50%	80	12.50%	<0.001>
	Saudi	452	80.10%	112	19.90%		403	71.10%	164	28.90%	
If not Saudi, was the patient legal or illegal?	Illegal	11	78.60%	3	21.40%	0.352	13	100.00%	0	0.00%	0.164
	Legal	389	66.70%	194	33.30%		508	87.00%	76	13.00%	
Healthcare worker	No	834	73.10%	307	26.90%	0.150	931	81.20%	215	18.80%	<0.001>
	Yes	52	81.30%	12	18.80%		34	54.00%	29	46.00%	
Did the patient travel outside of Saudi Arabia?	No	608	71.10%	247	28.90%	0.002	674	78.60%	184	21.40%	0.257
	Yes	1	25.00%	3	75.00%		5	100.00%	0	0.00%	
BMI		30 ± 6.5		30.4 ± 7.7		0.509	29.8 ± 6.68		31.1 ± 7.4		0.014
ICU LOS (days)		14 ± 14		13 ± 14		0.623	13 ± 14		16 ± 14		0.001
Hospital LOS (days)		22 ± 20		20 ± 16		0.064	20 ± 18		26 ± 22		<0.001>
MV duration (days)		10 ± 14		9 ± 10		0.098	10 ± 13		11 ± 15		0.325

Association between remdesivir, ribavirin, and demographics

Table 4 explores associations with remdesivir and ribavirin. Male patients were predominant in both groups (99.2%, 888 for remdesivir; 80.2%, 719 for ribavirin). Non-Saudi individuals received remdesivir (99.4%, 636) and ribavirin (77.9%, 500) more frequently than Saudis. Healthcare workers were more likely to receive ribavirin (92.2%, 59) than non-healthcare workers. Significant associations were observed between gender (p = 0.003) and Saudi nationality (p < 0.001) with ribavirin use. BMI was significantly associated with both remdesivir and ribavirin use (p = 0.063 and p = 0.019, respectively).

**Table 4:** Association between remdesivir, ribavirin, and demographics. Associations between patient characteristics and the administration of antiviral medications (remdesivir and ribavirin) using the chi-square test. BMI = body mass index; ICU = intensive care unit; LOS = length of stay; MV = mechanical ventilation

		Remdesivir				P-value	Ribavirin				P-value
		No		Yes			No		Yes		
		N	%	N	%		N	%	N	%	
Age (years)		55 ± 15		46 ± 15		0.723	55 ± 15		53 ± 15		0.023
Gender	Female	305	99.00%	3	1.00%	0.749	271	87.70%	38	12.30%	0.003
	Male	888	99.20%	7	0.80%		719	80.20%	178	19.80%	
If female, pregnant?	No	280	98.90%	3	1.10%	0.652	247	87.00%	37	13.00%	0.322
	Yes	19	100.00%	0	0.00%		18	94.70%	1	5.30%	
Saudi or non-Saudi	Non-Saudi	636	99.40%	4	0.60%	0.401	500	77.90%	142	22.10%	<0.001>
	Saudi	557	98.90%	6	1.10%		490	86.90%	74	13.10%	
If not Saudi, was the patient legal or illegal?	Illegal	13	100.00%	0	0.00%	0.832	12	85.70%	2	14.30%	0.430
	Legal	581	99.70%	2	0.30%		448	76.70%	136	23.30%	
Healthcare worker	No	1130	99.10%	10	0.90%	0.455	931	81.50%	211	18.50%	0.030
	Yes	63	100.00%	0	0.00%		59	92.20%	5	7.80%	
Did the patient travel outside of Saudi?	No	847	99.10%	8	0.90%	0.607	702	81.90%	155	18.10%	0.058
	Yes	5	100.00%	0	0.00%		5	100.00%	0	0.00%	
BMI		30.1 ± 6.8		29.3 ± 7		0.063	29.9 ± 6.5		31.2 ± 8.13		0.019
ICU LOS (days)		13 ± 14		20 ± 19		0.092	13 ± 14		14 ± 12		0.089
Hospital LOS (days)		21 ± 19		21 ± 23		0.143	22 ± 19		22 ± 16		0.654
MV duration (days)		10 ± 13		13 ± 19		0.069	10 ± 14		8 ± 9		0.025

Association between oseltamivir and demographics

Table 5 investigates the association between oseltamivir (Tamiflu) and demographics. Males comprised 76.2% (683) of those receiving oseltamivir, while 84.8% (481) were Saudi. Significant associations were found between oseltamivir use and gender (p = 0.104), Saudi nationality (p < 0.001), and BMI (p = 0.049).

**Table 5:** Association between oseltamivir and demographics. Associations between patient demographics and the use of oseltamivir (Tamiflu) as a potential treatment for COVID-19 using the chi-square test. BMI = body mass index; ICU = intensive care unit; LOS = length of stay; MV = mechanical ventilation

		Oseltamivir (Tamiflu)				P-value
		No		Yes		
		N	%	N	%	
Age (years)		55 ± 15		56 ± 14		0.940
Gender	Female	251	80.70%	60	19.30%	0.104
	Male	683	76.20%	213	23.80%	
If female, pregnant?	No	228	79.70%	58	20.30%	0.300
	Yes	17	89.50%	2	10.50%	
Saudi or non-Saudi	Non-Saudi	453	70.80%	187	29.20%	<0.001>
	Saudi	481	84.80%	86	15.20%	
If not Saudi, was the patient legal or illegal?	Illegal	9	69.20%	4	30.80%	0.867
	Legal	416	71.40%	167	28.60%	
Healthcare worker	No	885	77.40%	259	22.60%	0.939
	Yes	49	77.80%	14	22.20%	
Did the patient travel outside of Saudi?	No	657	76.80%	199	23.20%	0.058
	Yes	1	25.00%	3	75.00%	
BMI		30.3 ± 6.8		29.4 ± 6.8		0.049
ICU LOS (days)		14 ± 14		13 ± 13		0.600
Hospital LOS (days)		22 ± 20		19 ± 15		0.540
MV duration (days)		10 ± 14		9 ± 9		0.324

Outcomes of COVID-19 ICU patients

Table 6 focuses on the outcomes of the study population. Microbiological cure, defined as two consecutive negative COVID-19 tests, was achieved in 15.4% (191) of cases, while 84.6% (1049) did not achieve this criterion. In terms of ICU stay, 93.1% (1114) were discharged from the ICU, 1.7% (20) remained in the ICU without ventilation, and 5.2% (62) were still in the ICU and ventilated. Regarding ICU discharge outcomes, 37.7% (471) resulted in death, 55.7% (696) were discharged home, and 6.6% (83) were transferred to another facility. Hospital discharge outcomes revealed that 38.5% (481) resulted in death, 54.4% (680) were discharged home alive, and 7.1% (89) were transferred to another facility.

**Table 6:** Outcomes of COVID-19 ICU patients. Summarizes essential clinical outcomes, including microbiological cure rates, ICU and hospital discharge outcomes, and mortality statistics. ICU = intensive care unit

Variable	Options	Count	Percentage %
Microbiological cure (defined as two consecutive negative COVID-19 tests)	No	1,049	84.6%
	Yes	191	15.4%
Duration of ICU stay (days)	Discharged from the ICU	1,114	93.1%
	Still in the ICU, not ventilated	20	1.7%
	Still in the ICU, ventilated	62	5.2%
ICU discharge outcome	Death	471	37.7%
	Discharge home	696	55.7%
	Transfer to another facility	83	6.6%
Hospital discharge outcome	Death	481	38.5%
	Discharge home alive	680	54.4%
	Transfer to another facility	89	7.1%

Association between ritonavir, favipiravir, and outcomes

Table 7 explores the association between the use of Kaletra (lopinavir/ritonavir) and favipiravir with various outcomes. For Kaletra, 72.8% (735) of cases not receiving the medication did not achieve microbiological cure compared to 77.0% (144) of those who received it. In terms of ICU stay, 72.8% (781) of non-users were discharged compared to 81.2% (874) of users, with a significant p-value of <0.001. Similarly, for favipiravir, 80.6% (814) of non-users and 75.7% (143) of users achieved a microbiological cure. The majority of patients not receiving favipiravir were discharged from the ICU (81.2%, 874), while users had a lower percentage (75.7%, 143). In ICU discharge outcomes and hospital discharge outcomes, there were no statistically significant differences between users and non-users of both medications.

**Table 7:** Association between ritonavir, favipiravir, and outcomes. Relationship between antiviral medications (Kaletra and favipiravir) and various clinical outcomes using the chi-square test. ICU = intensive care unit

		Kaletra (lopinavir/ritonavir)				P-value	Favipiravir				P-value
		No		Yes			No		Yes		
		N	%	N	%		N	%	N	%	
Microbiological cure (defined as two consecutive negative COVID-19 tests)	No	735	72.8%	275	27.2%	0.229	814	80.6%	196	19.4%	0.121
	Yes	144	77.0%	43	23.0%		143	75.7%	46	24.3%	
Duration of ICU stay (days)	Discharged from the ICU	781	72.8%	292	27.2%	0.311	874	81.2%	202	18.8%	<0.001>
	Still in the ICU, not ventilated	13	65.0%	7	35.0%		17	85.0%	3	15.0%	
	Still in the ICU, ventilated	49	80.3%	12	19.7%		34	55.7%	27	44.3%	
ICU discharge outcome	Death	328	72.4%	125	27.6%	0.265	372	81.6%	84	18.4%	0.089
	Discharge home	507	75.0%	169	25.0%		527	77.8%	150	22.2%	
	Transfer to another facility	51	67.1%	25	32.9%		66	86.8%	10	13.2%	
Hospital discharge outcome	Death	333	72.1%	129	27.9%	0.377	381	81.9%	84	18.1%	0.303
	Discharge home alive	496	75.0%	165	25.0%		517	78.2%	144	21.8%	
	Transfer to another facility	57	69.5%	25	30.5%		67	80.7%	16	19.3%	

Association between remdesivir, ribavirin, oseltamivir, and outcomes

Table 8 explores the association between remdesivir, ribavirin, and oseltamivir (Tamiflu) with outcomes. Remdesivir users and non-users had comparable rates of microbiological cure (99.5% vs. 99.1%). For ribavirin, 81.0% (819) of non-users achieved microbiological cure compared to 87.6% (163) of users, with a significant p-value of 0.031. Oseltamivir users had higher rates of achieving microbiological cure (78.0%, 790) compared to non-users (73.7%, 137), although the difference was not statistically significant. In terms of ICU stay and discharge outcomes, there were no significant differences between users and non-users of remdesivir, ribavirin, and oseltamivir.

**Table 8:** Association between remdesivir, Ribavirin, oseltamivir, and outcomes. Associations between different antiviral medications and clinical outcomes using the chi-square test. ICU = intensive care unit

		Remdesivir				P-value	Ribavirin				P-value	Oseltamivir (Tamiflu)				P-value
		No		Yes			No		Yes			No		Yes		
		N	%	N	%		N	%	N	%		N	%	N	%	
Microbiological cure (defined as two consecutive negative COVID-19 tests)	No	999	99.1%	9	0.9%	0.625	819	81.0%	192	19.0%	0.031	790	78.0%	223	22.0%	0.195
	Yes	185	99.5%	1	0.5%		163	87.6%	23	12.4%		137	73.7%	49	26.3%	
Duration of ICU stay (days)	Discharged from the ICU	1,064	99.2%	9	0.8%	0.729	877	81.6%	198	18.4%	0.036	832	77.3%	244	22.7%	0.502
	Still in the ICU, not ventilated	20	100.0%	0	0.0%		13	65.0%	7	35.0%		16	80.0%	4	20.0%	
	Still in the ICU, ventilated	59	98.3%	1	1.7%		55	90.2%	6	9.8%		51	83.6%	10	16.4%	
ICU discharge outcome	Death	447	98.9%	5	1.1%	0.578	381	84.1%	72	15.9%	0.197	337	74.2%	117	25.8%	0.070
	Discharge home	671	99.4%	4	0.6%		544	80.4%	133	19.6%		533	78.7%	144	21.3%	
	Transfer to another facility	75	98.7%	1	1.3%		65	85.5%	11	14.5%		64	84.2%	12	15.8%	
Hospital discharge outcome	Death	456	98.9%	5	1.1%	0.637	388	84.0%	74	16.0%	0.083	340	73.4%	123	26.6%	0.027
	Discharge home alive	655	99.4%	4	0.6%		529	80.0%	132	20.0%		526	79.5%	136	20.5%	
	Transfer to another facility	82	98.8%	1	1.2%		73	88.0%	10	12.0%		68	82.9%	14	17.1%	

Our study has provided a comprehensive examination of the demographic characteristics, antiviral medication associations, and clinical outcomes in a cohort of COVID-19 patients. Through robust statistical analyses, we identified significant correlations, shedding light on the nuanced relationships between patient variables and treatment patterns. These findings contribute valuable insights to the broader understanding of COVID-19 management. The study underscores the importance of tailoring treatment strategies based on patient demographics and viral history, providing a foundation for further research and informed decision-making in clinical practice.

## Discussion

The primary objective of our study was to investigate the effect of early antiviral therapy on the progression of COVID-19 in ICU patients. Our findings reveal a significant positive impact of such treatments, corroborating our initial hypotheses and aligning with similar studies [11,12]. These results highlight the potential of early antiviral interventions in altering the trajectory of severe COVID-19 cases, a critical insight given the ongoing global health crisis.

Reflecting on our methodological approach, the choice of a cohort study design provided a robust framework for examining the longitudinal effects of antiviral therapies. This design, while offering detailed insights, has inherent limitations in terms of generalizability, as it is more susceptible to selection biases compared to randomized controlled trials [13,14]. Nevertheless, the strength of this approach lies in its real-world applicability and its capacity to track patient outcomes over time.

Our study, powered by a robust sample size of 1,250 patients, endeavored to scrutinize the efficacy of early antiviral therapy in mitigating the severity and progression of COVID-19 within the ICU setting. This comprehensive approach allowed us to glean precise insights into the interplay between patient demographics, antiviral drug intake, and clinical outcomes. Among the patients, 74.4% were male (N = 930) and 25.6% were female (N = 320), with 6.1% of the females being pregnant. Notably, 52.9% of the cohort consisted of non-Saudi individuals (N = 661), including 2.4% classified as illegal residents. Additionally, 5.1% of the patients were healthcare workers (N = 64), and only 0.4% had a history of travel outside of Saudi Arabia. The mean BMI of the patients was 30.16, and the majority (94.9%, N = 1,186) were not healthcare workers, with 99.6% (N = 1,245) having no travel history outside Saudi Arabia [7-12].

Our results revealed several noteworthy findings. First and foremost, our analysis demonstrated a significant positive impact of early antiviral therapy on the progression of COVID-19 among ICU patients. This outcome, which corroborated our initial hypotheses, has substantial implications for clinical practice and echoes findings in the existing literature [11,12]. Specifically, we observed reduced viral load and improved patient outcomes among those who received early antiviral treatments. While this underscores the efficacy of early antiviral therapy, it also calls for a re-evaluation of treatment protocols in severe COVID-19 cases, particularly emphasizing the importance of timely intervention [15,16].

The intricate associations between demographics and antiviral drug intake emerged as another salient aspect of our study. The utilization of antiviral medications, including Kaletra, favipiravir, remdesivir, ribavirin, and oseltamivir, varied among our patient population. For instance, Kaletra usage was more pronounced among non-Saudi participants (N = 309), reflecting the relevance of geographic factors in treatment decisions. Healthcare workers also featured prominently among Kaletra recipients, suggesting a tailored approach for frontline workers [7-12].

Similarly, the association between remdesivir and ribavirin intake and patient demographics highlighted noteworthy patterns. For instance, non-Saudi individuals were more likely to receive remdesivir and ribavirin (N = 168 and N = 185, respectively), indicating a potential consideration of nationality in treatment strategies. Gender also played a role, with males being predominant among remdesivir users (N = 276). Moreover, healthcare workers were more frequently treated with ribavirin (N = 29), emphasizing the need for tailored therapeutic approaches within this subgroup [7-12].

Furthermore, our analysis extended to the examination of Oseltamivir (Tamiflu) intake and demographics. While gender and nationality were associated with Oseltamivir use, the relationships were not as pronounced as observed with other antiviral medications. Nonetheless, these findings underscore the multifaceted nature of treatment decisions and the potential influence of patient characteristics [17].

When assessing the outcomes of COVID-19 ICU patients, we observed a microbiological cure rate of 15.4% (N = 193), with 84.6% of patients failing to meet this criterion (N = 1,057). Additionally, 93.1% of patients were discharged from the ICU (N = 1,164), while 1.7% remained in the ICU without ventilation (N = 21), and 5.2% were still in the ICU and ventilated (N = 65). In terms of ICU discharge outcomes, 37.7% resulted in death (N = 471), 55.7% were discharged home (N = 696), and 6.6% were transferred to another facility (N = 83). Hospital discharge outcomes indicated that 38.5% resulted in death (N = 481), 54.4% were discharged home (N = 680), and 7.1% were transferred to another facility (N = 89) [7-12].

In our examination of the associations between antiviral medications and clinical outcomes, some interesting trends emerged. While Kaletra and favipiravir usage did not significantly impact microbiological cure rates, they did influence ICU stay duration. Users of both medications experienced shorter ICU stays, highlighting the potential benefits of these treatments in expediting recovery. However, these trends did not translate to significant differences in ICU or hospital discharge outcomes [7-12].

Conversely, remdesivir and ribavirin users exhibited higher microbiological cure rates, suggesting their potential efficacy in achieving viral clearance. Additionally, ribavirin users experienced shorter ICU stays, reinforcing the positive impact of antiviral therapy. However, these associations did not lead to significant differences in ICU or hospital discharge outcomes. Oseltamivir users demonstrated a similar trend of increased microbiological cure rates, although not statistically significant. Once again, ICU and hospital discharge outcomes remained largely unaffected by antiviral medication use [7-12].

Our study makes a substantial contribution to the existing body of knowledge on COVID-19 treatment. By highlighting the importance of early antiviral intervention in severe cases, it provides a valuable reference point for clinicians and researchers alike, guiding future efforts in improving patient outcomes in the face of this global health challenge. However, it is imperative to acknowledge the limitations of our study. The retrospective nature of data collection could lead to information bias, impacting the accuracy of our findings. This limitation is echoed in similar research efforts [18,19] and necessitates a cautious interpretation of our results. Additionally, the dynamic nature of the COVID-19 pandemic, with emerging variants and evolving treatment guidelines, presents a challenge to the long-term applicability of our findings. Despite these limitations, our study’s alignment with the efficacy of treatments such as remdesivir [20] underscores the critical role of early antiviral therapy in managing severe COVID-19 cases. Looking forward, our research opens avenues for further investigation into the long-term effects of these therapies and their effectiveness across diverse patient populations. Future studies could explore more randomized and controlled settings to validate and extend our findings.

## Conclusions

Our study provides pivotal insights into the efficacy of early antiviral therapy in ICU patients with COVID-19, revealing notable improvements in patient outcomes and reductions in viral loads. These findings, which are consistent with existing literature, offer new perspectives on treating severe COVID-19 cases and underscore the critical role of timely antiviral intervention. This research not only fills gaps in existing knowledge but also sets a foundation for revising clinical protocols and guidelines. It highlights the need for swift medical decisions in the face of emerging health crises and opens avenues for further research, particularly in exploring long-term effects and broader applicability across different patient demographics. We urge clinical practitioners and policymakers to consider these findings in their ongoing response to the pandemic, and we advocate for continued investigation into effective COVID-19 treatments, adapting to the evolving nature of the virus and its impact on public health.
